# Molecular Predictors of Complete Response Following Neoadjuvant Chemotherapy in Urothelial Carcinoma of the Bladder and Upper Tracts

**DOI:** 10.3390/ijms20040793

**Published:** 2019-02-13

**Authors:** Jennifer Tse, Rashed Ghandour, Nirmish Singla, Yair Lotan

**Affiliations:** Department of Urology, University of Texas Southwestern Medical Center, Dallas, TX 75390, USA; jennifer.tse@phhs.org (J.T.); rashed.ghandour@utsouthwestern.edu (R.G.); nirmish.singla@utsouthwestern.edu (N.S.)

**Keywords:** urothelial carcinoma of the bladder, upper tract urothelial carcinoma, neoadjuvant chemotherapy, complete response, molecular markers

## Abstract

Urothelial carcinoma of the bladder (UCB) and upper tracts (UTUC) is often regarded as one entity and is managed generally with similar principles. While neoadjuvant chemotherapy (NAC) followed by radical cystectomy (RC) is an established standard of care in UCB, strong evidence for a similar approach is lacking in UTUC. The longest survival is seen in patients with complete response (pT0) on pathological examination of the RC specimen, but impact of delayed RC in nonresponders may be detrimental. The rate of pT0 following NAC in UTUC is considerably lower than that in UCB due to differences in access and instrumentation. Molecular markers have been evaluated to try to predict response to chemotherapy to reduce unnecessary treatment and expedite different treatment for nonresponders. A variety of potential biomarkers have been evaluated to predict response to cisplatin based chemotherapy including DNA repair genes (*ATM*, *RB1*, *FANCC*, *ERCC2*, *BRCA1*, and *ERCC1*), regulators of apoptosis (survivin, Bcl-xL, and emmprin), receptor tyrosine kinases (EGFR and erbB2), genes involved in cellular efflux (*MDR1* and *CTR1*), in addition to molecular subtypes (Basal, luminal, and p53-like). The current state of the literature on the prediction of response to NAC based on the presence of these biomarkers is discussed in this review.

## 1. Introduction

Urothelial carcinoma is the second most common genitourinary cancer in the United States, with an annual incidence for urothelial carcinoma of the bladder (UCB) and upper tract urothelial carcinoma (UTUC) of 81,190 and 3820, respectively [1]. The major cause of mortality is associated with presentation with muscle invasive (MIBC) or metastatic disease and most of these patients have high-grade disease. In 2003, level I evidence in favor of neoadjuvant chemotherapy (NAC) prior to radical cystectomy was published for UCB, although the benefit was modest [2]. In fact, the largest benefit was observed in complete responders to NAC with patients demonstrating no residual tumor (pT0) on radical cystectomy specimens. Patients with residual MIBC or nodal involvement after NAC are at high risk of recurrence and it is possible that patients who were nonresponders had progression of disease while receiving NAC. Identifying patients who are unlikely to respond could allow better selection of early cystectomy or enrollment in clinical trials. In UTUC, the benefit of NAC is not yet supported by randomized trials but there is evidence of potential benefit. In this review, we sought to summarize the available literature and evidence regarding the benefit of pT0 in both UCB and UTUC, as well as the clinical and molecular predictors for the complete response to NAC.

## 2. Prevalence and Prognosis of pT0

### 2.1. Urothelial Carcinoma of the Bladder

Although clinical stage is often used to guide treatments, there is often a discrepancy between clinical and pathologic staging, with pathologic upstaging and downstaging occurring in 42% and 22% of UCB patients from time of transurethral resection of the bladder tumor (TURBT) and subsequent RC, respectively [3]. Overall survival (OS) and other clinical outcomes after radical cystectomy (RC) are largely driven by pathologic stage. The problems with the current staging of bladder cancer are that imaging is poor at identifying micrometastatic disease and up to 25% of patients with MIBC have node positive disease at time of cystectomy. Also, it is difficult to identify invasion into perivesical fat during TUR or exam under anesthesia. An effort to identify risk factors for aggressive disease included clinical presence of hydroureteroneophrosis, lymphovascular invasion (LVI), and variant histological features (i.e., micropapillary or neuroendocrine) yet more than 50% of patients are understaged [4]. The reason some patients are downstaged is that TUR alone will sometimes eliminate all visible disease in the bladder and 5–15% of MIBC patients without NAC are found to have pT0 at cystectomy [2,5,6,7,8,9,10,11].

The recurrence-free survival (RFS) for MIBC after RC is 68% and 66% at 5 and 10 years, respectively, with most deaths occurring within the first three years attributable to bladder cancer [12]. Both RFS and OS are related to pathologic stage and lymph node status, with increasing stage portending a worse prognosis. While Stein et al. did not show a survival difference in subgroups of patients with organ-confined disease on cystectomy specimen (pTis/Ta, pT1, and pT2/T3a), these patients collectively had improved RFS and OS compared to extravesical tumors (5-year OS 85% vs. 58%) [12]. It is important to note that while most of this cohort underwent RC only, 10% received radiation, 5% received NAC, and 1% received a combination of NAC and radiation. Contemporary series show a 65% to 85% 5-year OS in patients who are pT0 at cystectomy [11,12,13].

Despite a markedly improved prognosis, not all patients with pT0 at RC are considered cured. In a multi-institutional cohort of patients undergoing RC between 1984 and 2003, Palapattu et al. analyzed the clinical outcomes of 59 pT0 patients, who accounted for 7% of their RC cohort [11]. Of these patients, 31 (55%) had muscle invasive disease on staging TUR, and neoadjuvant radiotherapy and chemotherapy were administered in 3% and 14% of these patients, respectively. Two patients (3%) had regional lymph node metastases. Five- and 10-year cancer specific survival (CSS) were 95% and 85%, respectively. Disease progression occurred in 6/59 (10%) of patients with RFS similar to those with pTa/Tis disease. This was also shown in a larger, more contemporary cohort (*n* = 228) of pT0 patients (56.2% of whom were cT2-4a) who did not receive preoperative chemotherapy and/or radiotherapy [5]. Despite no disease in the bladder, 17 (7.5%) had lymph node metastasis. Disease recurred in 23 patients (10.1%) including 14 patients who were lymph node-negative, suggesting some had micrometastatic disease that disseminated intravascularly prior to RC. Five and 10-year OS were 83.5% and 65.7%, respectively, similar to OS for pTa/Tis patients, but significantly improved compared to patients with >pT1 disease. On multivariate analysis, female gender and lymph node metastasis were associated with increased risk of disease recurrence and cancer specific mortality. Notably, clinical stage was not associated with survival, attesting to the poor correlation between clinical and pathologic stage.

### 2.2. Upper Tract Urothelial Carcinoma

UTUC accounts for almost 5% of urothelial malignancies and has an overall worse prognosis than UCB stage for stage. In a large multi-institutional series of 1363 patients who underwent radical nephroureterectomy (RNU) between 1992–2006, the 5-year RFS and CSS were 69% and 73%, respectively [14]. High tumor grade, increasing pathologic stage, lymph node metastases, sessile architecture, and lymphovascular invasion were independently associated with CSS. Prognosis of muscle invasive UTUC remains poor with 5-year CSS < 50% in pT2/T3 disease and less than 10% in pT4 disease [15]. With a mean follow-up of 51 months, 28% of patients had a disease recurrence [14]. It has been noted by Rink et al. that 80% of patients who experience a disease recurrence die within 2 years [16].

The reported rate of pT0 in patients undergoing radical nephroureterectomy (RNU) is between 0% and 0.7% [14,15,16,17,18]. This small number in comparison to UCB is likely due to the inability to completely resect or endoscopically ablate upper tract tumors due to the limitations of instrumentation and access through the ureter. While percutaneous resection of renal pelvis tumors is possible, it is rarely utilized in high grade tumors and may lead to spillage of tumor cells outside of the urinary tract. Yousef et al. noted an 85% 5-year CSS in pT0N0 patients which was statistically improved from those with residual disease (31%, *p* = 0.092) [17]. Nevertheless, the postoperative course of these patients is variable. In a combination of a French UTUC database and the UTUC Collaboration yielding 28 patients who were pT0 at RNU, four (14%) experienced extravesical disease recurrence, one in the surgical bed and three in distant sites, with a median time to recurrence of 38 months [19]. Those with metastatic disease died within a median of 10 months from the time of their disease recurrence. Nine (32%) additional patients developed intravesical recurrence within a median follow-up of 40 months. In this study, the 5-year recurrence-free and cancer-specific survival rates were 77% and 78%, respectively.

## 3. Factors Affecting Likelihood of pT0

One of the most important factors affecting pT0 rate in patients with organ confined disease is adequacy of TURBT. This is impacted by size, architecture, and location of the tumor and experience of the surgeon [20,21]. It is not clear if this is an important factor if the patient will proceed to RC but may have an impact in patients who pursue organ preservation with trimodal therapy [22]. Clearly, the patients with non-organ confined disease are unlikely to be cured regardless of the aggressiveness of TUR since disease in the perivesical fat or nodes cannot be impacted by TUR and there are risks associated with bladder perforation. However, it is important that a high-quality TUR is performed initially specially to ensure adequate staging of patients with MIBC.

### 3.1. Neoadjuvant Cisplatin-Based Chemotherapy in UCB

Multiple clinical trials have demonstrated that NAC improves OS in patients with MIBC. SWOG 8710 was a phase 3 clinical trial that randomized 317 patients with cT2-4 UCB over an 11-year period to undergo upfront RC or RC after receipt of three cycles of NAC with methotrexate, vinblastine, doxorubicin, and cisplatin (MVAC) [2]. The median OS was 77 months in patients who underwent NAC compared to 46 months in RC alone (*p* = 0.06). Of the 126 patients who received NAC, 48 (38%) exhibited complete pathologic response (ypT0), compared to 15% in the RC only group (*p* < 0.001). The five-year survival rate of pT0 patients was 85%, with a significantly higher median OS compared to those who had residual disease at RC (13.6 years vs. 3.4 years). In the BA06 30894 Trial by the Medical Research Council (MRC), 976 patients were randomized to NAC consisting of cisplatin, methotrexate, and vinblastine with radiotherapy (40%) or surgery or no NAC [23]. In the 211 patients who did not receive chemotherapy, 12.3% had no residual cancer on cystectomy sample compared to 67/207 (32.5%) who had received NAC. After a median follow-up of eight years, it was noted that there was a 16% reduction in the risk of death and 23% reduction in risk of metastases in patients who received NAC [24]. A meta-analysis of these trials demonstrated a 5% improvement in the 5 year survival rate with the addition of NAC compared to surgery alone although it was associated with substantial toxicity [25].

This toxicity, which includes neutropenia, infectious complications, and mucositis along with renal, cardiac, and neurologic toxicities in an already older patient population, has caused people to seek other chemotherapeutic options. In noninferiority trials, gemcitabine and cisplatin (GC) showed similar survival as MVAC with improved safety profile and no difference in the rate of pathologic downstaging [26,27,28,29]. An initial meta-analysis of clinical trials comparing MVAC and GC did show a reduced OS with GC (HR 1.26); however, when carboplatin, which is no longer recommended as a chemotherapy agent, was excluded, the difference was not statistically significant [30]. In addition, both dose-dense MVAC (ddMVAC) [31,32] and accelerated MVAC (aMVAC) [33,34] have been studied with similar outcomes and acceptable toxicity in comparison to traditional MVAC. In a recent study by Peyton et al. comparing ddMVAC to GC, there was a higher likelihood of downstaging and complete response with patients who received ddMVAC (pT0N0 41.3% vs. 24.5%) with a nonsignificant trend towards improved OS (HR 0.44, *p* = 0.16) [35].

Despite the guideline recommendations for the use of NAC in MIBC, its actual utilization remains low, although its use is increasing. Many barriers to routine use of NAC include the minimal perceived survival benefit, chemotoxicity, and delay to definitive extirpative therapy. Using the National Cancer Database, Zaid et al. noted that in a four year period, only 16.9% of patients who underwent RC for MIBC received NAC [36]. Factors associated with lower use of NAC included increasing age, lower patient income, and treatment at a nonacademic institution. A review of our own institutional experience of patients who underwent RC revealed a temporal increase in the utilization of cisplatin-based NAC from 17% (2003–2008) to 35% (2008–2012) [37].

The survival benefit afforded by the use of NAC is largely driven by the pathologic response, as reflected by the significantly higher rates of pT0 with NAC. Bhindi et al. evaluated patients who underwent NAC followed by RC with stage-matched controls (equivalent pT/pN stage, margin status, and year of RC) who underwent RC alone [38]. While the 5-year RFS and OS were not significantly different between NAC and control patients who were pT0, in patients with residual tumor at RC, those who received NAC had significantly worse 5-year RFS (50% vs. 64%) and OS (33% vs. 48%).

### 3.2. Clinical Predictors of Response to NAC

There are various clinical and pathologic factors that can impact the response to NAC. There is conflicting data regarding the significance of variant histologies in the treatment of UCB. AUA guidelines advocate consideration of early cystectomy in cT1 disease for patients with variant histology [39]. In addition, the evidence for the benefits of NAC in variant histology is unclear. While chemotherapy is recommended for patients with small cell MIBC, the benefits are unclear for micropapillary variant, and some advocate for upfront RC in patients with other pure histologic types (squamous, adenocarcinoma, sarcomatoid) [40]. In a multivariate analysis of patients who underwent NAC, Pokuri et al. analyzed whether age, histologic features, hydronephrosis, and chemotherapy type were predictors of complete response and found that only histologic type was significant, with pure urothelial carcinoma having greater odds of attaining pT0 compared to those with mixed histology (odds ratio 0.096) [7]. Focusing specifically at squamous differentiation, Minato et al. demonstrated that pathologic downstaging was significantly lower in patients with squamous differentiation compared to pure urothelial carcinoma (11.1% vs. 51.7%), which corresponded to worse RFS and OS [41]. In contrast, Scosyrev et al., who performed a secondary analysis of the SWOG 8710 trial and reported pathologic downstaging in 33/115 (29%) patients with pure urothelial carcinoma who received NAC compared to 11/32 (34%) patients with histologically mixed tumors after NAC [42]. Patients with mixed histology were found to derive benefit from NAC (HR 0.46, *p* = 0.02) with no difference in survival benefit from NAC when comparing mixed versus pure histology (HR 0.69, *p* = 0.14). In a meta-analysis of patients with micropapillary variant, NAC did result in pathological downstaging in a significant number of patients ranging from 11% to 55%, but this did not translate to better recurrence free survival outcomes [43].

Most patients diagnosed with UCB are initially non-muscle invasive (NMIBC) and are managed by TUR and intravesical therapy. Despite these interventions, ~30% of those with high grade NMIBC (Ta, Tis, T1) will progress to MIBC (i.e., secondary MIBC) [44]. Pietzak et al. studied the difference in clinical response in primary (*n* = 245) versus secondary MIBC (*n* = 43) [45]. On multivariate analysis, those with secondary MIBC had significantly worse RFS and OS compared to those with primary MIBC as well as a lower pathologic response (≤pT1) to NAC (26% vs. 45%, *p* = 0.02) with 0% of secondary MIBC patients obtaining a complete response. In a comparison of primary and secondary MIBC patients treated with NAC to those treated with RC alone, NAC increased the chance of pathologic downstaging in primary MIBC with improved RFS but worsened RFS in those with secondary MIBC. There is otherwise minimal data on clinical predictors of attaining pT0 specifically. Other factors to consider include the extent of TUR (though with increased risk of perforation), depth of invasion, and other tumor characteristics that may predict response to TUR or NAC [46].

### 3.3. Neoadjuvant Chemotherapy in UTUC

There is much interest in NAC for patients prior to nephroureterectomy (NU). A significant risk from cisplatin is nephrotoxicity and the main limitation to use is renal insufficiency. Patients have better renal function prior to NU and hence the impetus to give NAC before losing renal function. The likelihood of being able to receive any chemotherapy is greater prior to NU in the form of NAC than after NU as an adjuvant therapy. One of the other issues with NAC is that staging is very poor with ureteroscopy while grade is usually accurate. Giving NAC to all high-grade cancers will likely overtreat many patients with organ confined high grade UTUC.

There is currently no published level 1 evidence supporting the use of NAC in the management of high-grade UTUC. Much of our understanding of benefit from NAC in UTUC is extrapolated from data from UCB, retrospective studies, and small prospective cohorts at best. In a phase II study of three cycles neoadjuvant aMVAC of 10 patients with UTUC followed by RNU, 1/10 (10%) had a pathologic complete response and 4/10 (40%) were staged <pT1 [47]. Of note, only six patients completed all three cycles with others undergoing abbreviated cycles due to chemotoxicity. The combination of ddMVAC and bevacizumab was studied in a phase II clinical trial in 16 patients with UTUC [48]. Pathologic downstaging to ≤pT1 occurred in 75% of patients and complete response in 38% with a 2-year OS and DSS of 93% and 93% respectively. In retrospective analyses of NAC, patients generally received cisplatin-based chemotherapy (usually MVAC or GC) occasionally with ifosfamide [14,17,18,49]. In a meta-analysis of NAC, there appeared to be a DSS benefit with pooled HR of 0.41 in comparison to RNU only although more trials are ultimately needed to confirm utility [49]. We eagerly await the results of a multi-center prospective phase II trial of NAC followed by extirpative surgery for patients with high-grade UTUC, for which our institution is the lead site (clinicaltrials.gov NCT02412670).

In a comparison of biopsy-proven high-grade UTUC patients who underwent NAC followed by RNU (*n* = 43) with RNU only (*n* = 107), Matin et al. noted a significant reduction in pathologic stage in patients who received NAC despite the presence of higher risk factors [18]. Six (14%) had a complete pathologic response compared to 0% in the control cohort, and the overall incidence of patients with pT2 or higher disease was significantly decreased (46.5% vs. 65.4%, *p* = 0.43). The 5-year OS and CSS in the NAC group were 80.2% and 90.1% respectively compared to 57.6% and 57.6% in the RNU group. In a multivariate analysis in patients who were pN0, NAC had a significant influence on CSS and OS [50]. These findings were confirmed by Liao et al. who reported that 3 of 32 patients (9.4%) undergoing NAC achieved complete response [15]. The pathologic T stage was significantly lower in patients receiving NAC compared to those without NAC (37.5% vs. 59.6%). It is unclear whether a phase III trial will be performed to provide level 1 evidence of the benefit of NAC in UTUC.

## 4. Biomarkers Predictive of Response to Chemotherapy

### 4.1. The Need for Biomarkers

Although NAC has been shown to improve pathologic downstaging, only 30–40% of patients respond [29]. Consequently, in the current treatment paradigm many patients are exposed to the potentially toxic side effects of chemotherapy without much benefit. In fact, delaying RC for NAC in those patients has been shown to worsen their survival outcomes [51]. In recent years, there are several studies that evaluate biomarkers to help predict response to NAC. A major focus is related to response to cisplatin, which is the main active agent. Since cisplatin damages DNA, biomarkers associated with DNA repair have been studied most extensively. A summary of association of biomarkers and molecular subtypes with response to NAC based on the published studies is presented in Table 1 and Table 2.

### 4.2. DNA Repair Genes

Cisplatin acts as an alkylating agent, causing formation of DNA crosslinks and ultimately interfering with DNA replication. DNA damage induced by cisplatin is repaired by the nucleotide excision repair (NER) and homologous recombination (HR) pathways. The NER pathway involves multiple genes including *ERCC1-5*, *CDK7*, *DDB1-2*, *XPA*, and *XPC* [53]. *BRCA1*, *BRCA2*, and *RAD51* are involved in the HR pathway [63]. Germline changes in NER and HR pathways could affect the response to cisplatin-based chemotherapy.

Plimack et al. determined that alterations in one or more of three DNA repair genes, *ATM, RB1,* and *FANCC* not only predicted pathologic response but also better overall survival [52]. These alterations were predicted to be deleterious to the protein function. In the discovery cohort of 34 patients who were treated with MVAC, 13/15 (87%) of patients with a complete pathologic response (no residual MIBC, ≤pT1N0) had one or more alterations compared to 0% of nonresponders (*p* < 0.001). This was validated in a second cohort that underwent a different cisplatin-based chemotherapy regimen (GC) with 7/11 (64%) of responders exhibiting one or more alterations compared to 2/13 nonresponders (15%; *p* = 0.033). These alterations were also predictive of improved PFS and OS.

*ERCC2* (NER helicase) loss-of-function correlates with cisplatin sensitivity while overexpression causes cisplatin resistance [53]. Somatic *ERCC2* mutations have been identified in 12% of urothelial carcinomas in the TCGA [64]. In a prospective study by Van Allen et al., whole exome sequencing was performed on pre-chemotherapy tumor and germline DNA in 50 patients who received cisplatin-based NAC followed by RC (*n* = 25 pT0/pTis responders, *n* = 25 pT2+ nonresponders) [53]. *ERCC2* was the only gene significantly enriched in the responder cohort (36%) which was confirmed in two other bladder cancer populations. In vitro analysis of the *ERCC2* mutations suggests that they result in loss of normal NER capacity. These findings were confirmed in an independent validation cohort where 8/20 responders (40%) compared to 2/28 nonresponders (7%) had an *ERCC2* mutation [54]. Overall survival was statistically significant in both discovery and validation cohorts. In a comparison of primary and secondary MIBC, Pietzak et al. demonstrated that *ERCC2* missense mutations were more prevalent in primary MIBC (11% primary vs. 1.8% secondary, *p* = 0.044), which may account for the difference in pathologic downstaging between these two populations [45].

*BRCA1* is a tumor suppressor that helps maintain genomic stability by identifying DNA damage. *BRCA1* mRNA expression levels were measured in 57 patients with MIBC prior to NAC by Font et al. [56]. Median OS was 168 months in those with low/intermediate *BRCA1* levels compared to 34 months in those with high levels of expression and *BRCA1* levels were found to be an independent prognostic factor for OS on multivariate analysis. In addition, low/intermediate *BRCA1* levels were associated with a higher pathologic response to NAC compared to high levels (66% vs. 22%, *p* = 0.01). However, other studies have been unable to demonstrate any significant difference in OS based on level of *BRCA1* expression [57,63,65].

*ERCC1* is involved in excising bulky areas of DNA damage. Given that cisplatin-based chemotherapy leads to formation of these bulky adducts, increased *ERCC1* expression is more likely to cause cisplatin resistance and this has been shown in ovarian, gastric, cervical, colon, and non-small cell lung cancer [65]. The role of *ERCC1* in bladder cancer is more confusing since there is conflicting information about its role as a predictive marker and issues with its role as a prognostic marker. In a retrospective study of 57 patients with documented metastatic or locally advanced, surgical incurable (T4b, N0-1) urothelial carcinoma (including bladder and upper tract), patients with low levels of *ERCC1* mRNA expression had significantly longer median OS (25.4 months versus 15.4 months, *p* = 0.03) and increased time to disease progression than those with higher *ERCC1* expression [65]. Expression of *ERCC1* did not predict response to chemotherapy. Of note, only four (8.5%) patients underwent RC and response was measured by reevaluation of disease sites by physical examination, cystoscopy, and imaging. These results were again shown by Kim et al. in 89 patients with urothelial carcinoma (86.5% UCB, 13.5% UTUC) [57]. Forty-five per cent were *ERCC1*-negative and there was no difference in response rate to NAC between levels of *ERCC1* expression, although PFS, but not OS, was significantly longer in this group. While other studies [32,63] have not shown any correlation between *ERCC1* expression and pathologic response, which may be due to small sample size, Hemdan et al. reported significantly prolonged survival among patients with *ERCC1*-negative tumors who underwent NAC compared to observation (HR 1.77, *p* = 0.002), an improvement which was not identified in *ERCC1*-positive tumors [66].

*ERCC1* has also been studied in the adjuvant chemotherapy setting. Sun et al. retrospectively analyzed 93 patients who underwent RC without NAC, 61.3% of whom underwent adjuvant chemotherapy for advanced disease (T3/4 or N1) between three to eight weeks after RC [67]. *ERCC1*-positive status was identified in 58.1%, however this was not a prognostic factor for OS in the overall study population. In patients without adjuvant chemotherapy, *ERCC1-*-positive tumors had improved OS (84.0% vs. 49.2%) compared to *ERCC1*-negative patients; however, this effect was opposite in patients treated with adjuvant chemotherapy with a 5-year OS of 41.6% for *ERCC1*-positive tumors and 71.8% for *ERCC1*-negative (*p* = 0.074). It was suggested that adjuvant chemotherapy may have some potential benefit in *ERCC1*-negative tumors. In a larger cohort of 432 patients after RC, *ERCC1* positivity (71.3%) was associated with reduced disease recurrence than *ERCC1*-negative tumors (DFS 62% vs. 49%). Thirty-eight patients (8.7%) in this cohort underwent adjuvant cisplatin-based chemotherapy with no association of *ERCC1* with chemotherapy response. Currently, use of *ERCC1* as either a predictive or prognostic marker is unvalidated.

### 4.3. Regulators of Apoptosis

Chemotherapy relies on inducing apoptosis in its target cells. Consequently, antiapoptosis is a key factor in chemoresistance [68,69,70,71]. In MIBC, downregulation of members of antiapoptotic pathways, such as survivin, Bcl-xL, Rho-GDP dissociation inhibitor, tissue transglutaminase 2, and GADD45 are noted in long-term survivors [58]; si-RNA-mediated knockdown of Bcl-xl and survivin sensitized UCB cell lines to both mitomycin and cisplatin [72].

Retrospective studies have suggested that alterations in the tumor suppressor gene p53 are an independent prognostic factor for survival in patients treated with NAC, although pathologic response to NAC was not significant [73,74]. In a phase II study comparing neoadjuvant aMVAC and MVAC, *p53* were not associated with complete pathologic response [33]. In addition, the type of mutation in p53 did not affect response. Similarly, in a phase III trial by Stadler et al., patients with pT1/T2N0M0 disease with tumors demonstrating >10% nuclear reactivity on immunohistochemistry for p53 (considered altered) were offered three cycles of adjuvant MVAC or observation [75]. There was no difference in RFS or OS between patients with p53 positive tumors (55% of cohort) versus p53 negative tumors, nor any survival difference in p53 positive patients who underwent chemotherapy. Of note, there was a high refusal rate and poor compliance in the adjuvant chemotherapy arm and it could be argued that this specific cohort is typically not the cohort studied for adjuvant chemotherapy.

Als et al. used Affymetrix GeneChip expression profiling to identify prognostic markers in locally advanced and/or metastatic UCB patients following cisplatin-based chemotherapy [58]. A set of 55 genes with a high correlation to survival time was identified, including emmprin and survivin. These were then validated using immunohistochemistry in a separate cohort of 124 patients receiving cisplatin-based chemotherapy. Emmprin, a modulator of matrix metalloproteinases, is upregulated in UCB compared to benign urothelium and has been shown to enhance growth and resistance to chemotherapy [76,77]. Survivin inhibits apoptosis by inhibiting activation of caspases. High levels of survivin have been associated with poor prognosis in UCB [78]. Low levels of emmprin and survivin expression significantly correlated with OS both separately and in combination. In addition to these factors, the presence or absence of visceral metastases was an independent prognostic factor for OS. In patients without visceral metastases, the median survival time was 21.2 months. In this subgroup, median survival time was increased to 47.3 months if the tumor was negative for both emmprin and survivin, decreased to 17.5 months if the tumor was negative for only one of the two proteins, and reduced even further to 6.6 months if the tumor was positive for both (*p* < 0.0001). The response to chemotherapy was 82% if the tumor was negative for both emmprin and survivin.

### 4.4. Receptor Tyrosine Kinase

Epidermal growth factor receptor (EGFR) is expressed in the basal layer in normal urothelium. Overexpression has been reported in urothelial carcinoma, most evident in MIBC [79]. Erb-b2 receptor tyrosine kinase 2 (erbB2) is highly upregulated in NMIBC and MIBC compared to normal tissue [80]. In an analysis of 178 cancer-associated genes in prechemotherapy TUR specimens by Groenendijk et al., *ERBB2* had the highest enrichment for mutations in complete responders compared to nonresponders [55]. In their discovery and validation cohort combined, *ERBB2* missense mutations were seen in 9/38 (24%) complete responders as compared to 0/33 (0%) of nonresponders (*p* = 0.003). Half (5/10) of the identified missense mutations were located at amino acid 310 (S310), which is a mutational hotspot associated with activating mutations. *ERBB2* amplification was not associated with response to NAC (*p* = 0.52). 

### 4.5. Genes Involved in Cellular Efflux

*MDR1* encodes for an integral membrane protein, Pgp, that acts as an energy-dependent cellular efflux pump and has been shown to reduce the intracellular concentrations of a number of cytotoxic drugs resulting in blunted chemotherapeutic response [81]. Petrylak et al. demonstrated that treatment with MVAC led to a significantly increased expression of *MDR1* [82]. The AUO-AB 05/95 trial compared adjuvant cisplatin and methotrexate (CM) to methotrexate, vinblastine, epirubicin, and cisplatin (MVEC). The investigators found that CM was not inferior to MVEC with regard to RFS, but resulted in significantly less grade 3 and 4 leukopenia [83]. Hoffman et al. analyzed RNA from tumor samples (*n* = 108) in this trial cohort for the expression of *MDR1* [81]. Their analysis demonstrated that patients with a lower level of *MDR1* expression had a higher chance of survival and a lower risk of progression. Five-year survival was only 25% in patients with high *MDR1* compared to 62% in those with low expression. In addition, after two years, 65% of those with high *MDR1* expression had progressed compared to only 25% of patients with low *MDR1* expression. On multivariate analysis, vascular invasion was the strongest independent risk factor for OS followed by high levels of *MDR1* expression and pN2 stage. *MDR1* expression was a stronger determinant of patient outcomes in the MVEC arm compared to the CM arm.

Copper transporter receptor 1 (CTR1), an uptake transporter of copper, plays a role in the influx of platinum (Pt), modulating intracellular Pt concentration. In a retrospective review of patients who underwent cisplatin-based NAC and RC, Kilari et al. determined that high expression of CTR1 on TUR specimens correlated with pathologic downstaging to NMIBC (*p* = 0.0076) [59]. In 43% of patients, the CTR1 tumor expression level was maintained between pre- and post-chemotherapy specimens while 40% of patients experienced an upregulation of CTR1 in response to chemotherapy. All remaining patients had downregulation of CTR1 expression and were noted to have muscle invasive disease at the time of cystectomy, although the numbers were too small to make any meaningful conclusions.

### 4.6. Gene Expression Profiling and Molecular Subtypes

Molecular subtypes with distinct molecular profiles can potentially complement pathologic classification and aid in clinical decision making and counseling. Choi et al. performed whole genome mRNA expression profiling in a cohort of fresh-frozen MIBCs obtained after TUR [60]. Three molecular subtypes were identified and described as basal, luminal, and p53-like, which are similar to prior subtype classifications [84]. The basal subtype is characterized by p53 activation, positive CK5/6, high levels of EGFR, lack of cytokeratin 20, and enrichment with sarcomatoid and squamous features. Patients with these tumors were more likely to present with metastatic disease and exhibit shorter CSS and OS. Despite this, immune-infiltrated basal tumors respond to NAC, suggesting that more aggressive early management of this subtype may offer the best chance for improved survival. Luminal tumors exhibited strong peroxisome proliferator-activated receptor (PPAR) pathway and estrogen receptor (ER) activation as well as activating FGFR3 mutations. Luminal tumors did appear to respond well to NAC (4/6 in discovery cohort), although no specific biomarkers for chemosensitivity could be determined. p53-like tumors contained an activated wild type p53 gene expression signature. In the discovery cohort, all p53 tumors (*n* = 7) were resistant to NAC. Even in an expanded NAC cohort (*n* = 34) and an additional cohort of 23 tumors treated with MVAC, p53-like tumors remained resistant to NAC. Furthermore, in comparing pre- and post-treatment gene expression profiles of chemoresistant tumors (even those previously characterized as basal or luminal), chemotherapy caused all tumors to express an active p53 pathway gene signature after NAC, although this signature was distinct from the p53 signature within the subtype group.

These subtypes were further analyzed by McConkey et al. in urothelial carcinoma (both bladder and upper tract) who underwent neoadjuvant ddMVAC with bevacizumab followed by surgery [61]. Pathologic pT0N0 rate was 39% and 38% in UCB and UTUC, respectively, with downstaging to ≤pT1N0 occurring in 53% of patients, which significantly improved CSS and OS. Tumor subtype on TUR tissue (*n* = 38) was subdivided into basal (*n* = 11), luminal (*n* = 11), and p-53-like (*n* = 16). Basal tumors were noted to have better survival in comparison to luminal and p53-like subtypes (5-year OS: 91%, 73%, and 36%, respectively) which was confirmed in a separate cohort. Bone metastasis within 2 years only occurred in those with the p53-like subtype. Subtypes at cystectomy did not correlate with survival and there was a shift to the p53-like subtype at cystectomy (basal to p53-like, 60%; luminal to p53-like, 71%). UTUC was more likely to be of the luminal subtype. The difference in outcome for basal subtype in comparison to the prior study may reflect the heterogeneous sample size which included patients who did not undergo NAC. Interestingly, subtype at TUR was not significantly associated with downstaging after NAC.

In addition to the above subtypes, a claudin-low subtype was described by Kardos et al. [85]. These tumors arise primarily from basal tumors but show significantly increased rates of *RB1*, EP300, and NCOR1 mutations as well as increased percentage of EGFR mutations and decreased FGFR3 mutations. In addition, they are highly enriched in immune gene signatures and express high levels of immune checkpoint molecules. Seiler et al. ultimately developed a single sample genomic subtyping classifier (GSC) to predict consensus subtypes (claudin-low, basal, luminal-infiltrated, and luminal) in the context of NAC [62]. In regard to OS, the GSC basal tumors had a 3-year OS rate of 49.2% in the non-NAC cohort compared to 77.8% in the NAC cohort. This pronounced difference was not shown in luminal tumors and both luminal-infiltrated and claudin-low tumors responded poorly regardless. However, GSC was not significantly associated with a major pathological response to NAC (<pT2). In a comparison to NAC responders and nonresponders in each subtype, patients with luminal tumors that experienced a major response had an improved OS compared to nonresponders (95% vs. 58%), but basal tumors did not exhibit any significant difference in OS. The authors suggested that NAC had the greatest impact on OS for basal tumors but may not be as beneficial for those with the luminal subtype given their already favorable prognosis. They urged alternative therapies for claudin-low and luminal-infiltrated subtypes.

### 4.7. Future Directions

Ultimately, the goal of identifying biomarkers and molecular subtypes would be to individualize cancer treatment and predict each patient’s pathologic response to NAC and overall clinical outcome. A gene expression algorithm—“coexpression extrapolation” (COXEN)—combines molecular profiling of cancer cells lines and drug sensitivity that could be translated into predicting clinical response [86]. Chemosensitive biomarkers are identified in cell lines from NCI-60, which is composed of cell lines from diverse human cancers (excluding UCB) and a model is created to predict drug response in a separate cohort of cell lines or tumors based on their gene expression. We eagerly await the results of SWOG 1314, a randomized phase II trial to test the ability of the COXEN algorithm to predict the efficacy of neoadjuvant cisplatin-based therapy in UCB, which is expected to be completed in 2020 [87]. In this study, patients are randomized to GC or MVAC chemotherapy and their gene and microRNA expression collected prior to chemotherapy. The COXEN algorithm will be used to determine whether it accurately predicts their response to chemotherapy. This model may allow us to stratify patients, determining who would benefit the most from NAC compared to upfront cystectomy and their optimal chemotherapeutic regimen.

## 5. Cost Effectiveness Analysis

Incorporating biomarkers into current clinical decision-making could potentially predict response to NAC and avoid chemotherapy in patients who are unlikely to respond, thus expediting surgery and decreasing the cost and toxicity associated with NAC. We developed a decision analysis model to evaluate the cost effectiveness of a biomarker-based approach to NAC using a DNA repair gene panel (*ATM*, *RB1*, and *FANCC*), *ERCC2*, and RNA subtypes [88]. Our model compared RC alone, unselected NAC followed by RC, and biomarker-directed NAC followed by RC. We found that the DNA repair gene panel-based approach was the most cost-effective strategy with a mean OS of 3.14 years, $31,482/life year. Under this model, 38% of patients would go on to receive NAC. This approach increased mean OS by 5.2 months and 1.6 months compared to RC alone and compared to a scenario where all patients received NAC.

## 6. Conclusions

Invasive urothelial carcinoma is associated with a high mortality rate; however, increasing use of NAC has continued to improve the OS in this population. Complete pathologic response (pT0 stage) is a strong predictor of OS and likely drives the primary benefit observed from the administration of NAC in MIBC. We currently lack level 1 evidence that demonstrates the benefit of NAC in the setting of UTUC, however the approach offers advantages since patients have better renal function than after nephroureterectomy. Achieving pT0 status is difficult in UTUC due to limitations of instrumentation and access to the upper urinary tract. Unfortunately, many patients with urothelial carcinoma do not respond to platinum-based therapy. This results in unnecessary toxicity and delay of definitive therapy which can negatively impact their survival. The use of biomarkers to stratify NAC administration based on predicted response may be a cost-effective option, although prospective validation is needed before this approach would be ready for primetime.

## Figures and Tables

**Table 1:** Summary of association of biomarkers with response to NAC based on the published studies.

Ref	Biomarker	Patients (*n*)	Definition of Response	Results		
				Marker Pos/Responder (%)	Marker Pos/Nonresponder (%)	*p*-Value
[52]	*ATM*, *RB*, *FANCC*	Discovery: 34 Validation: 24	≤pT1N0M0	13/15 (87%) 7/11 (64%)	0/19 (0%) 2/13 (15%)	<0.001 0.033
[53]	*ERCC2*	50	pT0/Tis	9/25 (36%)	0/25 (0%)	<0.05
[54]		48	pT0/pTis/pTa	8/20 (40%)	2/28 (7%)	0.01
[55]		71	pT0	6/38 (16%)	2/33 (6%)	0.27
[56] ^1^	*BRCA1*	57	≤pT1N0M0	24/28 (85%)	15/29 (52%)	0.01
[57] ^2^	*ERCC1*	89	≤pT1N0M0	34/61 (56%)	15/28 (54%)	0.62
[33]	*P53*	39	pT0	7/14 (50%)	12/25 (48%)	NS
[58] ^3^	Emmprin, Survivin	124 Emmprin Survivin Combination	Not defined	20/75 (27%) 24/74 (32%) 6/42 (14%)	30/49 (61%) 28/50 (56%) 16/24 (67%)	<0.05 <0.05 <0.05
[55]	*ERBB2*	71	pT0	9/38 (24%)	0/33 (0%)	0.003
[59]	*CTR1*	44	≤pT1N0M0 pT0	13/20 (65%) 4/7 (57%)	6/24 (25%) 15/37 (41%)	0.0076

^1^ Low/intermediate levels of BRCA1 were associated with pathologic response to NAC. ^2^ *ERCC1* negative patients had a prolonged RFS (10.6 mo vs. 8.4 mo, *p* = 0.03). ^3^ Emmprin and survivin-negative tumors were associated with improved response. Of note, combination category compares patients who were either positive or negative for both emmprin and survivin. NS: non-significant.

**Table 2:** Summary of association of molecular subtypes with response to NAC based on the published studies.

Ref	Molecular Subtype	Patients (*n*)	Definition of Response	Response Number (%)	*p*-Value
[60] Discovery	Basal Luminal P53-like	5 6 7	pT0 or pT1	2 (40%) 4 (67%) 0 (0)	0.018
[60] Philadelphia Validation	Basal Luminal P53-like	14 20 9	pT0 or pT1	7 (50%) 12 (60%) 1 (11%)	-
[61]	Basal Luminal P53-like	11 11 16	≤pT1N0M0	5 (45%) 8 (73%) 5 (31%)	-
[62] ^1^	Claudin-low Basal Luminal-infiltrated Luminal	57 68 33 111	≤pT1N0M0	23 (40%) 28 (41%) 9 (27%) 48 (43%)	NS

^1^ Basal and luminal-infiltrated tumors who showed a response unfortunately did not have an improved survival. Claudin-low and luminal tumor subtypes did show an improved overall survival. NS: non-significant
